# *Mycobacterium tuberculosis* Rv0229c Shows Ribonuclease Activity and Reveals Its Corresponding Role as Toxin VapC51

**DOI:** 10.3390/antibiotics12050840

**Published:** 2023-05-01

**Authors:** Sung-Min Kang

**Affiliations:** College of Pharmacy, Duksung Women’s University, Seoul 01369, Republic of Korea; smkang@duksung.ac.kr

**Keywords:** toxin–antitoxin system, *Mycobacterium tuberculosis*, VapC51, ribonuclease activity

## Abstract

The VapBC system, which belongs to the type II toxin–antitoxin (TA) system, is the most abundant and widely studied system in *Mycobacterium tuberculosis*. The VapB antitoxin suppresses the activity of the VapC toxin through a stable protein–protein complex. However, under environmental stress, the balance between toxin and antitoxin is disrupted, leading to the release of free toxin and bacteriostatic state. This study introduces the Rv0229c, a putative VapC51 toxin, and aims to provide a better understanding of its discovered function. The structure of the Rv0229c shows a typical PIN-domain protein, exhibiting an β1-α1-α2-β2-α3-α4-β3-α5-α6-β4-α7-β5 topology. The structure-based sequence alignment showed four electronegative residues in the active site of Rv0229c, which is composed of Asp8, Glu42, Asp95, and Asp113. By comparing the active site with existing VapC proteins, we have demonstrated the justification for naming it VapC51 at the molecular level. In an in vitro ribonuclease activity assay, Rv0229c showed ribonuclease activity dependent on the concentration of metal ions such as Mg^2+^ and Mn^2+^. In addition, magnesium was found to have a greater effect on VapC51 activity than manganese. Through these structural and experimental studies, we provide evidence for the functional role of Rv0229c as a VapC51 toxin. Overall, this study aims to enhance our understanding of the VapBC system in *M. tuberculosis*.

## 1. Introduction

*Mycobacterium tuberculosis* has been persistent in humankind, even in harsh environments, for a long era [1]. Often, *M. tuberculosis* shows tolerance to survival threats such as antibiotic treatments [2]. It can re-activate quite some time after infection and cause disease. Because of its persistence, the treatment of tuberculosis may take a relatively long time [3,4,5]. *M. tuberculosis* has evolved a genetic system to combat many practical events such as temperature variability, nutrient depletion, and other environmental assaults [6]. Thus, the eradication of this persistent bacteria remains a big challenge.

In a typical toxin–antitoxin (TA) system, the gene encoding toxin and the gene encoding antitoxin share a same operon and are located adjacent to each other [7]. Accordingly, the transcription and translation of the toxin and antitoxin occur concurrently to maintain an adequate stoichiometric ratio between the toxin and the antitoxin [8]. The toxin exerts deleterious activity and the antitoxin neutralizes the toxin’s activity. TA systems act as addiction modules that confer maintenance and stability in bacteria such as *M. tuberculosis* [9].

Although there have been many biochemical studies in this field, the biological role of the TA system is still a difficult subject of much debate. TA systems are a bicistronic operon, comprised of a toxin and an antitoxin. The toxin targets essential cellular processes such as cell wall synthesis, cell division, and DNA replication, leading to cell growth arrest and in some cases cell death [10]. Depending on how the antitoxin regulates the activities of toxins, TA systems can be divided into several different classes [11]. The absence of continuous expression of antitoxin or degradation of antitoxin can release free toxin. TA systems have been reported to be implicated in various functions including pathogenesis, virulence, persistence, biofilm formation, survival from stress, and multidrug tolerance [12].

VapC51, the subject of this paper, belongs to the type II TA system. *M. tuberculosis* has a remarkably expanded number of type II TA systems and the most abundant is the virulence-associated protein (VapBC) system [13]. In type II TA systems, the toxins are stable proteins, and the cores of these proteins commonly feature an antiparallel β-sheet [14]. The antitoxins are also proteins, usually having loose and flexible structures; thus, they are prone to degradation [15]. In healthy conditions, the antitoxin suppresses the toxin’s activity through formation of a stable protein–protein complex. However, when cells suffer environmental stresses, the balance between toxin and antitoxin is broken [16]. The antitoxin degrades by stresses; the remaining free toxin is released to act on their substrates, driving the cells to the bacteriostatic state [17]. Similarly, VapB antitoxin and VapC toxin constitute the VapBC system, belonging to type II TA systems [18]. VapC toxins contain a PilT *N*-terminal (PIN) domain that can cleave cellular RNA [19]. The active sites of VapC toxins contain several conserved acidic residues that form a negatively charged cavity, which coordinates divalent metal ions such as Mg^2+^ and Mn^2+^ [19,20]. VapB antitoxins consist of two functional motifs: an *N*-terminal domain that binds to the promoter DNA of the TA operon and a C-terminal domain that binds to the cognate VapC toxin to neutralize its toxicity [20].

This communication aims to provide an additional understanding of the VapC toxins by introducing the six known structures. They are *M. tuberculosis* VapC2 [21], VapC5 [22], VapC11 [23], VapC15 [24], VapC26 [25], and VapC30 [26]. The overall structures of the six VapC toxins display a sandwich-like topology and feature a compact α/β/α main domain that is composed of a four-stranded parallel β-sheet surrounded by α-helices. In terms of function, VapC5 toxin has been experimentally shown to possess ribonuclease activity in a nuclease assay, and in vitro tests have demonstrated its ability to cleave general 150 nucleotide RNA [22]. In addition, the VapC15 toxin exhibited catalytic activity in degrading RNA from *Escherichia coli* in an in vitro ribonuclease activity assay using agarose gel electrophoresis [24]. In a study on VapC26 and VapC30, both toxins demonstrated the ability to cleave RNA substrates in an in vitro ribonuclease assay. [25,26].

Here, we are introducing the Rv0229c, a putative VapC51 toxin, classified in the VapBC family most recently. Using structural and experimental studies, we address the conformation and function of Rv0229c. To provide a broader understanding of *M. tuberculosis* VapC toxin, we identify the supposed role of Rv0229c as VapC51 toxin based on biophysical and biochemical knowledge.

## 2. Results and Discussion

### 2.1. Overall Structure

**Figure 1:**
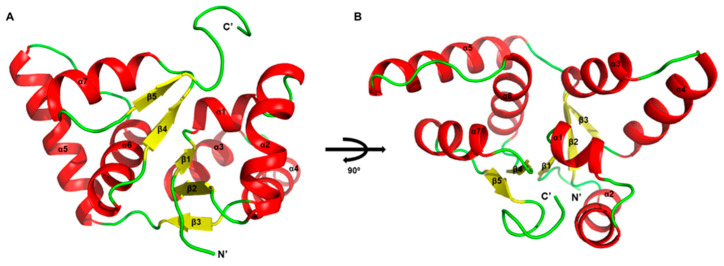
Structure of *M. tuberculosis* Rv0229c. Structure of Rv0229c monomer in two different orientations. (**A**) Overall architecture of Rv0229c. The α helices in an asymmetric unit are shown in red; β strands are shown in yellow; residual loops are shown in green. (**B**) 90° rotated view of (**A**).

We attempted to determine the structure of Rv0229c, but it was challenging to attain crystallizable conditions. Therefore, we have decided to replace the description of the overall structure with one derived from Alphafold 2 (Figure 1A,B). Because Alphafold 2 is not perfectly accurate when predicting oligomeric states, we mainly conducted structural analysis on the monomeric form. The Rv0229c molecule is a globular protein with a tightly packed structure that resembles a typical PIN-domain protein, exhibiting an α/β/α sandwich topology [27,28]. The sandwich comprises of five beta strands arranged in parallel and surrounded by four alpha helices at the *N*-terminal and three at the C-terminal. The order of the secondary structure is β1-α1-α2-β2-α3-α4-β3-α5-α6-β4-α7-β5. The structure that has been rotated 90° resembles a bird spreading its wings.

### 2.2. Structural Comparison

**Figure 2:**
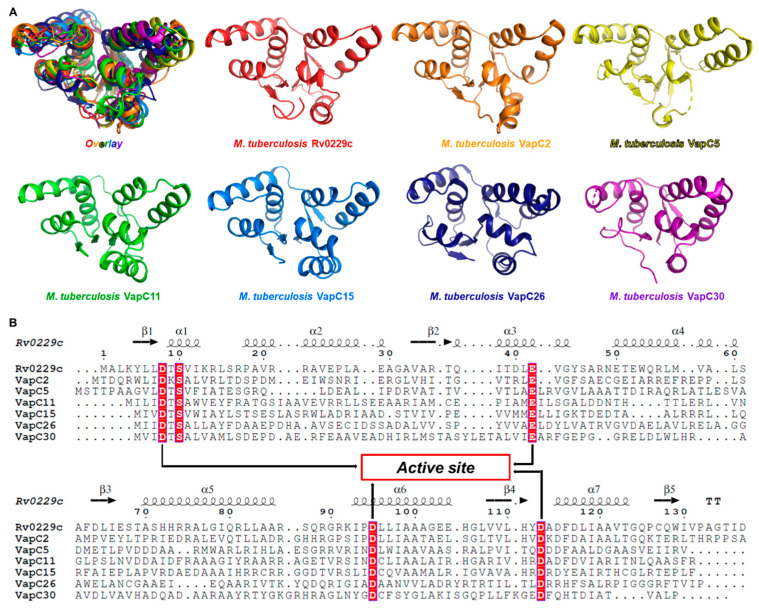
Structural and sequential alignments of known *M. tuberculosis* VapC structures. (**A**) Frontal views of *M. tuberculosis* VapC structures in rainbow colors including Rv0229c (red); PDB ID 3H87 (VapC2, orange); PDB ID 3DBO (VapC5, yellow); PDB ID 6A7V (VapC11, green); PDB ID 4CHG (VapC15, blue); PDB ID 5X3T (VapC26, indigo); PDB ID 4XGR (VapC30, purple). (**B**) Sequential alignments of Rv0229c and *M. tuberculosis* VapC structures. Secondary structural information of Rv0229c is provided in first line. Highly conserved residues are shown in red. Arrows represent the confirmed conserved active site residues. Active site residues show high conservation.

Compared to other non-pathogenic mycobacteria, *M. tuberculosis* exhibits a significantly higher number of Type II VapBC TA systems [29]. The VapBC family is characterized by the toxin (VapC), which has a PilT *N*-terminal (PIN) domain with an RNase-H-like fold and nuclease activity [30]. To initially determine if Rv0229c corresponds to the VapC toxin, a structural alignment was performed with the support of sequence alignment (Figure 2A). The results of the structural comparison between Rv0229c and other VapC proteins showed: the sequence identity with *M. tuberculosis* VapC2 (PDB ID 3H87) was 41%, the z score was 19.3, and the rmsd was 1.9 Å [21]; the sequence identity with *M. tuberculosis* VapC5 (PDB ID 3DBO) was 22%, the z score was 14.1, and the rmsd was 2.5 Å [22]; the sequence identity with *M. tuberculosis* VapC11 (PDB ID 6A7V) was 26%, the z score was 17.5, and the rmsd was 2.2 Å [23]; the sequence identity with *M. tuberculosis* VapC15 (PDB ID 4CHG) was 19%, the z score was 18.5, and the rmsd was 1.8 Å [24]; the sequence identity with *M. tuberculosis* VapC26 (PDB ID 5X3T) was 18%, the z score was 13.9, and the rmsd was 2.2 Å [25]; the sequence identity with *M. tuberculosis* VapC30 (PDB ID 4XGR) was 20%, the z score was 13.9, and the rmsd was 2.5 Å [26].

The results of structure-based sequence alignment revealed four clear electronegative active site residues (Figure 2B). The core residues comprising three aspartate residues and one glutamate residue (Asp8, Glu42, Asp95, Asp113) form the active site and contribute to Rv0229c being a VapC toxin with ribonuclease activity. Based on these findings, it can be confirmed that it is reasonable to identify Rv0229c as VapC51.

### 2.3. Active Site

In *M. tuberculosis*, approximately ~100 type II toxin–antitoxin systems have been identified, with the majority falling under the VapBC family of ribonucleases, which consists of more than 50 members (VapC1 to VapC51 or more) [31]. These VapC toxins share a common PIN catalytic domain that contains 4–5 acidic residues that are crucial to the functioning of the toxin as they coordinate one or more divalent cations in the catalytic center [32].

**Figure 3:**
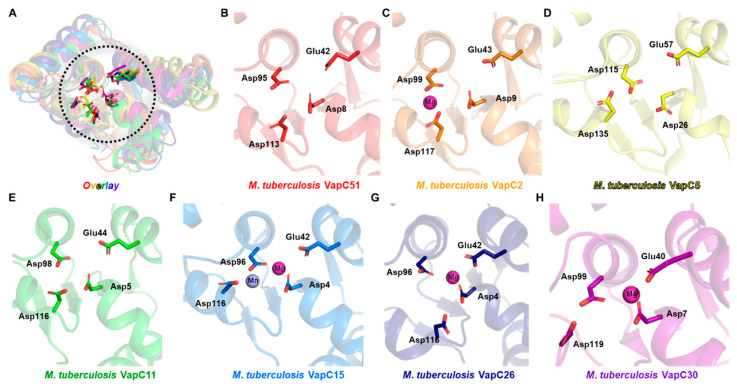
Active sites of VapC51 and other *M. tuberculosis* VapC structures. (**A**) Structural superposition of VapC51 homologs. Active sites are enclosed in the black dotted circle. Conserved active site residues are shown in ball and stick presentation. Scrutinized views of active sites are shown in (**B**–**F**). Some structures lack metal ions. Colors used are the same as Figure 2. (**B**) *M. tuberculosis* VapC51 (AF-L0T5V6-F1); (**C**) *M. tuberculosis* VapC2 (PDB ID 3H87); (**D**) *M. tuberculosis* VapC5 (PDB ID 3DBO); (**E**) *M. tuberculosis* VapC11 (PDB ID 6A7V); (**F**) *M. tuberculosis* VapC15 (PDB ID 4CHG); (**G**) *M. tuberculosis* VapC26 (PDB ID 5X3T); (**H**) *M. tuberculosis* VapC30 (PDB ID 4XGR).

The comparison of the active site architecture with homologs revealed that the first three residues (Asp8, Glu42, Asp95) show a high degree of conservation, but the fourth residue (Asp113) has significant deviation among homologs (Figure 3A–H). Although not all homolog structures have a divalent metal, magnesium or manganese were observed in structures with divalent metals. Thus, it can be predicted that the activity of VapC51 will be regulated by divalent metals such as magnesium or manganese.

As shown by the results of structural comparison, although VapC toxins share structural similarity in their active sites, they are known to recognize different targets [33,34]. While the target specificities of all VapC toxins used in the comparison are not known, it is reported that *M. tuberculosis* VapC11 and VapC15 cleave tRNA and VapC26 cleaves rRNA [33]. Interestingly, among the VapC toxins used in the comparison, VapC26 has significant deviation in the location of the fourth active site residue (Asp116) compared to VapC11 (Asp116) and VapC15 (Asp116). This suggests that the location of the relatively less conserved fourth active site residue may also determine the substrate specificity, and it can be predicted that VapC51, similar to VapC11 and VapC15, cleaves tRNA.

### 2.4. Ribonuclease Activity

**Figure 4:**
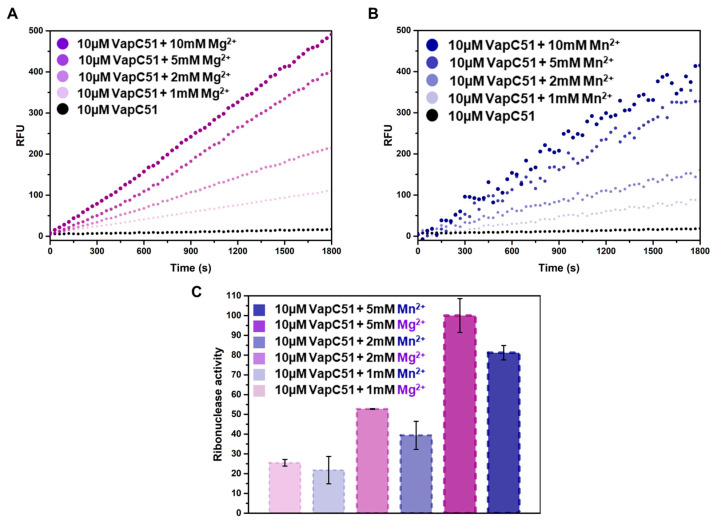
Fluorescence-based in vitro assays with VapC51, Mg^2+^, and Mn^2+^. (**A**) Relative fluorescence units (RFU) of 10 µM VapC51 with the addition of Mg^2+^ (0 mM, 1 mM, 2 mM, 5 mM, and 10 mM). (**B**) Relative fluorescence units (RFU) of 10 µM VapC51 with the addition of Mn^2+^ (0 mM, 1 mM, 2 mM, 5 mM, and 10 mM). (**C**) Data for 1 mM, 2 mM, and 5 mM metal added in (**A**) and (**B**) are plotted as the mean of ± standard deviation (SD) of the results of three independent replicates in triplicate.

To determine the role of magnesium and manganese on the activity of VapC51, an in vitro ribonuclease activity assay was performed. In this experiment, the concentration of VapC51 was fixed at 10 µM, and the concentrations of magnesium and manganese were adjusted from 1 mM to 10 mM (Figure 4A,B). By comparing the relative fluorescence unit (RFU) values of multiple samples, which are essentially the amount of light collected by the instrument, we can determine the relative intensity of fluorescence. The results indicated that the RFU was not significantly different when the metal concentration was 5 mM or 10 mM, suggesting that most enzymatic activity was saturated at a metal concentration of 5 mM. In addition to comparing kinetics over time, quantitative analysis was also performed to compare the effects of magnesium and manganese, revealing that magnesium contributes more significantly to the activity of VapC51 than manganese (Figure 4C). Fluorescence was hardly detected when only protein was present without metal ions, or when substrate RNA and metal ions were added without protein. To prevent contamination from any free RNase that may exist in the environment, 40 units of RiboLock™ (Thermo Scientific, Waltham, MA, USA) Rnase inhibitor were added to all reaction wells.

## 3. Materials and Methods

### 3.1. Cloning and Transformation

The gene encoding the Rv0229c was amplified by *M. tuberculosis* H37Rv whole genomic DNA. The used primers in PCR were 5′-GGAATTCCATATGGCGCTGAAATATCTT-3′ and 5′-TTACCGCTCGAGGTCGATCGTGCCCGCTGG-3′ as the forward and reverse, respectively. A plasmid for Rv0229c was prepared using pET28a vector. The amplified DNA of Rv0229c was inserted into and pET28a vector. Restriction enzymes used for ligation were *Nde1* and *Xho1*. The resulting ligated construct contains *N*-terminal hexa-histidine tag (MGSSHHHHHHSSGLVPRGSH). Recombinant plasmid was transformed into *Escherichia coli* DH5α competent cells and verified by DNA sequencing. For expression, the recombinant plasmid was transformed into *E. coli* C41 competent cells. These cells were grown in Luria broth (LB) medium supplied with kanamycin (40 µg/mL) at 37 °C.

### 3.2. Protein Expression and Purification

Expression of recombinant *N*-terminal hexa-histidine tagged Rv0229c protein was induced by the supplement of 0.5 mM isopropyl β-D-1-thiogalactopyranoside (IPTG) upon arriving at OD_600_ value of 0.6 and the cell culture was further grown at 37 °C for an additional 4 h, respectively. These cells were harvested by centrifugation at 8000× *g* at 4 °C. The cell pellet was resuspended with lysis buffer *A* (50 mM Tris–HCl pH 7.5, 500 mM NaCl) and ultra-sonication was applied. Supernatant of resulting lysate was completely cleared by centrifugation at 22,000× *g* for 1 h at 4 °C. The cleared supernatant of Rv0229c was inserted into an Ni^2+^–nitrilotriacetate (Ni–NTA) affinity column (Sigma Aldrich, St. Louis, MO, USA) and eluted with elution buffer containing imidazole (150–450 mM). To remove imidazole, further size-exclusion chromatography was conducted by a HiLoad 16/60 Superdex 200 preparatory-grade column (GE Healthcare, Chicago, IL, USA) in an experimental buffer (20 mM Tris–HCl, pH 7.5 and 150 mM NaCl). The purified proteins were analyzed for their purity by SDS–PAGE and concentrated to 500 µM stock solution by ultrafiltration in 10 kDa molecular-mass cutoff spin columns (Millipore, Burlington, NJ, USA).

### 3.3. Structural Information

Crystal screening of purified Rv0229c was conducted using the commercial kits; crystal screening 1, 2 and Index 1, 2 (Hampton Research, Aliso Viejo, CA, USA)/Structure screen 1, 2, 3, 4 (Rigaku Reagents, Bainbridge Island, WA, USA). However, crystals of sufficient quality to obtain diffraction data for structural determination were not produced. Thus, alternately, the predicted three-dimensional structure of VapC51 (Rv0229c) was exploited from the AlphaFold 2 Protein Structure Database (AF Database, London, UK) [35]. The entry of the computed structure model of ribonuclease VapC51 is AF-L0T5V6-F1 and global pLDDT score was 96.6.

### 3.4. Bioinformatics

Structural figures were generated using PyMol (The PyMOL Molecular Graphics System, Version 2.5, Schrödinger, LLC). Superimpositions of structures were performed with the secondary-structure matching (SSM) [36] option within Coot [37]. The quality of the fitted structures was qualified at the wwPDB X-ray structure validation server [38]. The rmsd and Z score were calculated by Dali and Multiprot server complementarily [39,40]. Genome data were utilized from the KEGG and UniProt database [41,42]. Structure-assisted alignments were carried out using ESPript [43] with the help of ClustalW [44] for sequence alignment.

### 3.5. In Vitro Ribonuclease Activity Assay

To confirm the ribonuclease activity of the VapC51, fluorescence-based ribonuclease activities of VapC51 with Mg^2+^ and Mn^2+^ were examined. Protein was pre-treated with ethylene-diamine-tetra-acetic acid (EDTA) to be unbound from any metal. We prepared synthetic RNA substrate which has random sequences (IDT). Specific fluorophore is covalently attached to one edge of a synthetic RNA and the quencher group is located at the other edge. When this synthetic RNA meets a ribonuclease such as VapC51, the quencher becomes detached and we can observe fluorescence at 520 nm upon excitation at 490 nm. In this assay, the concentration of VapC51 was fixed at 10 µM, whereas the concentrations of Mg^2+^ and Mn^2+^ were varied from 1 mM to 10 mM. The concentrations of proteins were identified by Nanodrop (Sigma-Aldrich) and the relative fluorescence unit (RFU) value was observed by a SPECTRAmax GEMINI XS spectrofluorometer.

## 4. Conclusions

In this study, we focused on Rv0229c from *M. tuberculosis* and identified it as VapC51 based on the structural and functional evidence. We achieved an understanding on the functional implications of the active site architecture of the PIN domain, as well as the regulatory role of magnesium and manganese on its ribonuclease activity. Additionally, further studies can be performed to understand the biological function in *M. tuberculosis*, including its role in the bacterial response to stress and its potential as a drug target.

If antibiotic drugs are developed based on the VapBC systems of *M. tuberculosis*, artificially activating VapC toxins can be a powerful antibacterial strategy [45,46]. Normally, VapB and VapC form a tightly bound TA complex, which keeps the toxin protein inactive. However, under environmental stress, the antitoxin is separated and the toxin is activated, leading to bacterial death. Therefore, compounds that interfere with the binding of the toxin and the antitoxin can be used as a strategy to kill bacteria by utilizing the mechanism of bacterial cell apoptosis. This method uses the inherent toxicity of the bacteria, which may result in fewer side effects in humans, and can selectively regulate only certain pathogenic bacteria containing specific VapBC systems.

Given the high number of VapBC TA systems in *M. tuberculosis*, exploring the exact functions between different VapC toxins will also provide important insights into the pathogenesis of tuberculosis. Therefore, further research is needed to fully understand the mechanisms and functions of the VapBC51 system, including its antitoxin component VapB51, which serves to neutralize the toxin and sustain the cellular stability of bacteria.

## Data Availability

No new data were created or analyzed in this study. Data sharing is not applicable to this article.
